# Machine learning classifier approaches for predicting response to RTK-type-III inhibitors demonstrate high accuracy using transcriptomic signatures and *ex vivo* data

**DOI:** 10.1093/bioadv/vbad034

**Published:** 2023-03-22

**Authors:** Mauricio H Ferrato, Adam G Marsh, Karl R Franke, Benjamin J Huang, E Anders Kolb, Deborah DeRyckere, Douglas K Grahm, Sunita Chandrasekaran, Erin L Crowgey

**Affiliations:** University of Delaware, Newark, DE 19716, USA; University of Delaware, Newark, DE 19716, USA; Nemours Children Health System, Wilmington, DE 19803, USA; Department of Pediatrics, University of California San Francisco, San Francisco, CA 94143, USA; Helen Diller Family Comprehensive Cancer Center, University of California San Francisco, San Francisco, CA 94143, USA; Nemours Children Health System, Wilmington, DE 19803, USA; Department of Pediatrics, Emory University School of Medicine, Atlanta, GA 30322, USA; Department of Pediatrics, Emory University School of Medicine, Atlanta, GA 30322, USA; University of Delaware, Newark, DE 19716, USA; Nemours Children Health System, Wilmington, DE 19803, USA

## Abstract

**Motivation:**

The application of machine learning (ML) techniques in the medical field has demonstrated both successes and challenges in the precision medicine era. The ability to accurately classify a subject as a potential responder versus a nonresponder to a given therapy is still an active area of research pushing the field to create new approaches for applying machine-learning techniques. In this study, we leveraged publicly available data through the BeatAML initiative. Specifically, we used gene count data, generated via RNA-seq, from 451 individuals matched with *ex vivo* data generated from treatment with RTK-type-III inhibitors. Three feature selection techniques were tested, principal component analysis, Shapley Additive Explanation (SHAP) technique and differential gene expression analysis, with three different classifiers, XGBoost, LightGBM and random forest (RF). Sensitivity versus specificity was analyzed using the area under the curve (AUC)-receiver operating curves (ROCs) for every model developed.

**Results:**

Our work demonstrated that feature selection technique, rather than the classifier, had the greatest impact on model performance. The SHAP technique outperformed the other feature selection techniques and was able to with high accuracy predict outcome response, with the highest performing model: Foretinib with 89% AUC using the SHAP technique and RF classifier. Our ML pipelines demonstrate that at the time of diagnosis, a transcriptomics signature exists that can potentially predict response to treatment, demonstrating the potential of using ML applications in precision medicine efforts.

**Availability and implementation:**

https://github.com/UD-CRPL/RCDML.

**Supplementary information:**

Supplementary data are available at *Bioinformatics Advances* online.

## 1 Introduction

The leukemia and lymphoma society (LLS) has established a novel infrastructure for treating blood cancers through the BeatAML initiative. This approach relies on a collaborative concept for utilizing personalized genomic data in the context of matching subjects to targeted treatment strategies (Leukemia and Lymphoma Society, 2021). Recently, Tyner *et al.* (2018) published a comprehensive overview of the functional genomic landscape of samples associated with the BeatAML initiative. In these efforts, they conducted extensive *ex vivo* drug testing, genomic sequencing and characterization of patient responses to various therapeutic approaches. Over the years, it has proven challenging to implement targeted therapies for acute myeloid leukemia (AML), due to the complexities of genomic alterations associated with this blood cancer and a high relapse occurrence (Estey *et al.*, 2015).

In recent years, machine learning (ML) techniques have been used as a potential solution for identifying or distinguishing responders versus non-responders, thereby helping to address precision medicine problems pertaining to complex diseases such as AML (Eckardt *et al.*, 2020). For example, gene expression data and the regression algorithm LASSO have been used for classification of AML versus other cancers for over 12 000 subjects across 105 different studies (Warnat-Herresthal *et al.*, 2020). However, regarding classification algorithms (CA) for predicting response to therapy, a scalable ML approach has not been efficiently identified. Recently, investigators have reported on support vector machines (Castillo *et al.*, 2019; Gal *et al.*, 2019; Lee *et al.*, 2018), k-nearest neighbor (Castillo *et al.*, 2019; Gal *et al.*, 2019; Lee *et al.*, 2018), RF (Castillo *et al.*, 2019; Gal *et al.*, 2019), artificial neural networks (Janizek *et al.*, 2021) and gradient boosting (Janizek *et al.*, 2021) for conducting these types of analysis.

In our research, we focused on using ensemble algorithms (RF and gradient boosting that also include LightGBM) as classifiers. Due to the high-dimensional structure of the data, identification of gene markers is an important focus in the field as researchers look for ways to reduce training time and minimize overfitting. Many feature extraction algorithms, or rather feature selection techniques, for gene expression data have been proposed, from the traditional use of principal component analysis (PCA; Gal *et al.*, 2019) to customized techniques (Castillo *et al.*, 2019; Lee *et al.*, 2018). Some of these works include the EXPRESS (Janizek *et al.*, 2021) algorithm that uses ensembles of XGBoost models and the Shapley Additive Explanation (SHAP) package to calculate a global feature importance ranking. Yap *et al.* (2021) used SHAP to explain a convolutional neural network to classify samples on 47 different tissues based on RNA-seq count data and compared the genes identified by SHAP with differential expression analysis. Johnsen *et al.* (2021) developed a new method where they combine tree-ensemble methods (XGBoost) and SHAP to identify potential (Single Nucleotide Polymorphisms – SNPS) SNP–SNP interactions and tested it by analyzing the importance ranking generated by SHAP from the trained classifiers using a UK Biobank dataset. Hathaway *et al.* (2019) used SHAP in combination with Classification and Regression Trees to classify type-2 diabetic patients versus non-diabetic patients based on cardiac physiological, biochemical, genomic and epigenomic biomarker data. For this study, we focused on classifying patients as responders versus non-responders, benchmarking a combination of three different feature selection techniques and three classifiers, and demonstrated that the SHAP feature selection technique combined with a tree-based ensemble classifier is able to predict with high accuracy a responder versus a non-responder using RNA-seq and *ex vivo* drug response data.

## 2 Methods

### 2.1 The BeatAML dataset

The dataset utilized for this project was provided in the original manuscript (Burd *et al.*, 2020) and consists of the RNA-seq count data and *ex vivo* drug response data. The RNA sequence dataset contains gene counts count per million (CPM) of 22 844 genes for 451 clinical patients (Supplementary Table S1). The drug response data matrix contains the area under the curve (AUC; from receiver operator characteristic curves [ROCs]) and ic50 scores for samples tested on 122 different drug inhibitors (Supplementary Table S2). There are total of 528 overall samples, where each inhibitor contains different subsets of patients. These drug subsets range in size from 100 to 510 patients.

### 2.2 Drug response distributions

**Fig. 1.**
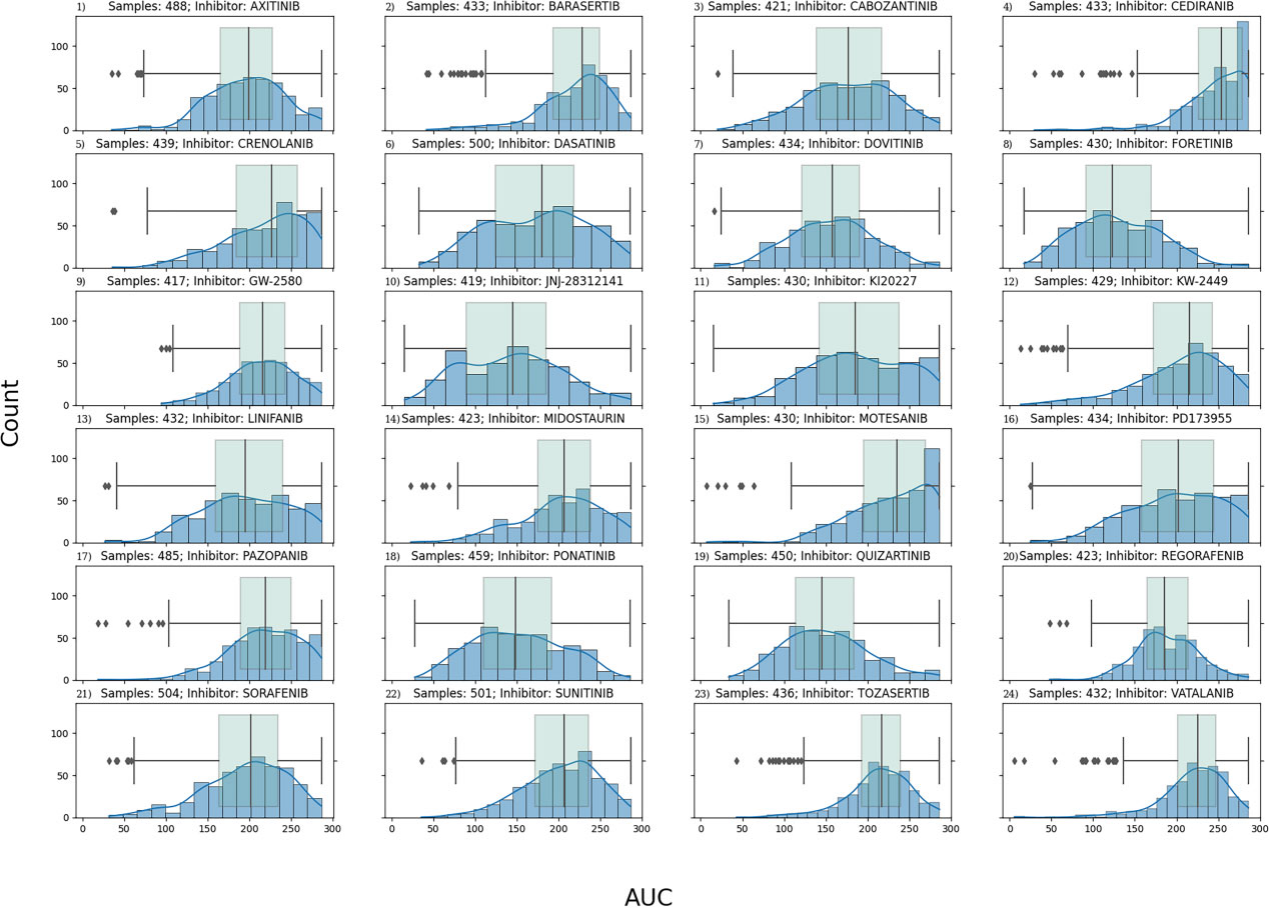
Quartile analysis for responder versus non-responder. This figure shows the drug response AUC distribution of the 24 RTK-type-III inhibitors in the form of blue histogram plots. Overlaid on top of the histogram is also green box-whisker plot of the same distribution. Each plot is titled with the name of its given inhibitor. The *y*-axis is the drug response AUC score for the histogram bin, and the *x*-axis is the count, or number of samples that fall in the bin. We can see that some of the distributions are skewed. The ones skewed towards the left correspond to drug therapies where most patients responded positively, whereas the ones skewed towards right are for drug therapies where most patients did not respond. Some drug therapies have a normal distribution where there is no disproportion of patients that responded well or not. By focusing on the tails of the distribution by looking at the box-whisker, the patients can be classified as low/high responders depending on which tail (low → right, high → left) side they fall into

For each of the drugs analyzed, a distribution plot of the AUC and ic50 values was generated. For each distribution, the upper and lower quartiles were determined, and subjects were divided into responder (>0.75 quantile) versus non-responder (<0.25 quantile). These were the labels assigned to each subject for classification purposes. Any subjects whose drug response fell between the first and third quartiles were not assigned to a response group and therefore not included in our responder versus non-responder analyses. Figure 1 contains all the AUC distributions for the 24 drugs analyzed. A table that includes the number of samples per label (responder versus non-responder) used for each model developed is available as Supplementary Table S3. All data handling and preprocessing were done using the pandas (v1.3.5; McKinney, 2010; The Pandas Development Team, 2021) python package.

### 2.3 ML workflow

**Fig. 2.**
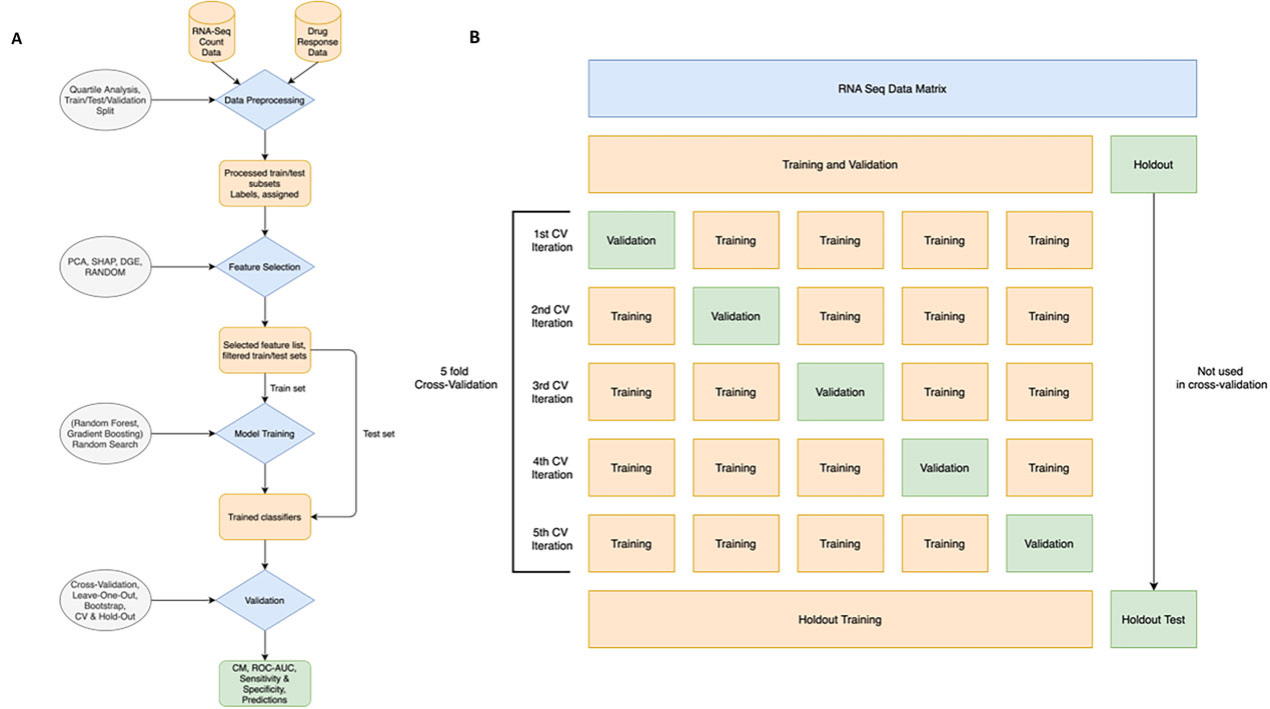
Overview of the RCDML pipeline. (**A**) The RCDML pipeline is broken down into four main processing steps (diamonds): data preprocessing, feature selection, model training and validation. For each step, there is an input and an output (square). The descriptions (ovals) are indicated per each corresponding workflow step. The final output of the model consists of a confusion matrix that indicates the number of false positive, false negatives, true positives, and true negatives. (**B**) The training, validation, and test dataset splitting are shown here. The original dataset is split into two sets, training and validation and the holdout dataset. The training and validation set is split into five folds, where one of the folds at each iteration is used for validation. After the model is selected, the training and validation set is used for training the selected model and the holdout dataset is used for testing

Collectively, the ML workflow consisted of the following steps: (i) data preprocessing, (ii) feature selection, (iii) classifier training and (iv) validation (Fig. 2A).

#### 2.3.1 Data preprocessing

To reduce the complexity of our initial work with the BeatAML data, we focused our analyses on the RTK-type-III family of drug inhibitors that had at least 300 patient observations, which yielded 24 drug inhibitors, patient subsets (Supplementary Table S4). For each inhibitor, samples were matched between the drug response data and the RNA-seq data to identify the available samples of each experiment.

#### 2.3.2 Training, validation and test dataset split

The dataset is randomly shuffled and split into two sets: Phase 1—80% of the dataset, used for model optimization in a 5-fold cross-validation (CV) approach. Phase 2—20% of the dataset, which is hidden from the model, and used as the holdout/test. In Phase 1, the dataset is broken down into five training/test pairs, and each of these pairs is used as input for the next steps in the workflow. The model evaluates all possible samples available as it iterates through each fold. The model with the highest AUC score during Phase 1 (training and test data) is selected for Phase 2, when the holdout dataset is used as inference to evaluate the model selected. An overview of this process is shown in Figure 2B.

#### 2.3.3 Feature selection

After the training set is established, our pipeline performs feature selection to identify a set of ‘likely’ informative gene markers (classification signature) that each model will use to produce a response categorization. For this workflow, three different feature reduction approaches were selected: Shapley values (SHAP), differential gene expression (DGE) analysis and PCA.

The SHAP (Lundberg and Lee, 2017) feature selection technique was implemented using the SHAP package python and consisted of using the TreeExplainer algorithm on a trained XGBoost model to generate Shapley values using the shap_values() functions. When the Shapley values are generated, each sample has a list of marginal contribution values that explain its output. To acquire the ‘global’ Shapley value of the model, the absolute mean of these marginal contributions is taken across the feature axis. Then, once we have individual values for each feature, the values are sorted from highest to lowest, and the top features are selected.

Using R statistical package LIMMA (v3.50.1; Ritchie *et al.*, 2015) a DGE analysis was conducted between responders versus nonresponders (see Fig. 1 for quartile description). Genes which did not have at least 1 CPM across either the responder or non-responder group were removed before statistical testing. A false discovery rate (FDR) threshold of 0.05 was used. Ensemble transcript ids were mapped to a gene name using the UniProt.org (UniProt Consortium, 2015) mapping tool.

The PCA technique was conducted using the implementation found in the scikit-learn (v1.0.2; Pedregosa *et al.*, 2011) python package. The fit_transform() function is used to fit the PCA technique using the training data and applying the dimensionality reduction on it, then transform() is used to apply the decomposition on the test data. The resulting matrices are then sliced to select number of features.

The training set passes through one of these approaches and 30 features are selected. This number was chosen because it would be smaller than the sample size of the training folds for all inhibitor models. The features selected represent the best candidates for classification based on the technique’s algorithm approach. The filtered output is used as the training set for the classifiers.

#### 2.3.4 Classifier training

For the classifiers, three ensemble-based algorithms were utilized: RF (scikit-learn v1.0.2; Pedregosa *et al.*, 2011; Ho, 1995) and two implementations of the Gradient Boosting algorithm (LightGBM v3.3.3; Ke *et al.*, 2017) and (XGBoost v1.5.2; Chen and Guestrin, 2016). A random search hyperparameter approach was used where different parameter combinations were tested using a 5-fold CV approach, and the best-performing parameter list (the ones with higher ROC–AUC scores) was selected. The parameter lists for each classifier are available in Table 1. In Phase 2, the classifiers with the best-performing parameters selected are used to classify the holdout samples.

**Table 1.** Parameter list used to optimize the RF and gradient boosting classifiers

Classifier	List of parameters
RF (scikit-learn)	‘bootstrap’: [True, False], ‘max_depth’: [10, 20, 30, 40, 50, 60, 70, 80, 90, 100, 110, None], ‘max_features’: [‘auto', ‘sqrt’], ‘min_samples_leaf’: [1, 2, 4], ‘min_samples_split’: [2, 5, 10], ‘n_estimators’: [100, 150, 200, 250, 500, 750, 1000]
Gradient boosting (XGBoost)	‘max_depth’: [10, 20, 30, 40, 50, 60, 70, 80, 90, 100, 110, None], ‘learning_rate’: [0.001, 0.01, 0.1, 0.2, 0.3], ‘subsample’: [0.5, 0.6, 0.7, 0.8, 0.9, 1.0], ‘colsample_bytree’: [0.4, 0.5, 0.6, 0.7, 0.8, 0.9, 1.0], ‘colsample_bylevel’: [0.4, 0.5, 0.6, 0.7, 0.8, 0.9, 1.0], ‘min_child_weight’: [0.5, 1.0, 3.0, 5.0, 7.0, 10.0], ‘gamma’: [0, 0.25, 0.5, 1.0], ‘reg_lambda’: [0.1, 1.0, 5.0, 10.0, 50.0, 100.0], ‘n_estimators’: [100, 150, 200, 250, 500, 750, 1000]
Gradient boosting (LightGBM)	‘max_depth’:[10, 20, 30, 40, 50, 60, 70, 80, 90, 100, 110, None], ‘learning_rate’: [0.001, 0.01, 0.1, 0.2, 0.3], ‘subsample’: [0.5, 0.6, 0.7, 0.8, 0.9, 1.0], ‘colsample_bytree’: [0.4, 0.5, 0.6, 0.7, 0.8, 0.9, 1.0], ‘min_child_weight’: [0.5, 1.0, 3.0, 5.0, 7.0, 10.0], ‘reg_lambda’: [0.1, 1.0, 5.0, 10.0, 50.0, 100.0], ‘n_estimators’: [100, 150, 200, 250, 500, 750, 1000]

#### 2.3.5 Validation

At each inference operation, the validation dataset (Phase 1) or the holdout dataset (Phase 2) is used to calculate confusion matrices, sensitivity and specificity. Using these scores, the model generates ROC–AUC curves. This is generated for each fold of the 5-fold CV approach, and the results presented are the average of the results across the CV folds as well as for the holdout model. An AUC score that is higher than 0*.*50 means that the model had some predictive power when assigning the samples into groups, with higher scores corresponding to higher sensitivity and specificity scores.

### 2.4 Experimental setup

The code ran on the DARWIN cluster at the University of Delaware. The specification for the node is CPU—AMD EPYC 7002 Series, 32 Cores (64 total dual-socket), Memory—512 GB RAM. The list of software and versions used for the Python and R environments is available as Supplementary Table S5.

### 2.5 Statistical analysis for analyzing the ML approach

All statistical procedures were performed using R (v4.1) as defined scripts executed on either OSX or Linux (Ubuntu) platforms. The first phase of compiling the dataframes for analysis used outlierTest (package car), shapiro.test for assessing normality (package stats) and the levene.test() for homogeneity of variance (package lawstat). Analysis of the final dataframes used standard parametric procedures via anova() for analysis of variance (ANOVA) and Tukey honestly significant difference (HSD) test for identifying significant mean differences (both in package stats).

Initial distributions of AUC values were non-normal across feature reduction tools (FRT) that include DGE, PCA and SHAP by CA such as RF, XGBoost and LightGBM. A large source of variance was the large difference in response rates (AUC) from different drug inhibitors. Because we know that each drug is different—different mode of action), different metabolic phenotype targets, thus different genotypes—we focused on within a drug, how well does each FRT perform to identify target transcript pattern differences. In essence, we approached each drug as a paired or block variable and expressed AUCs as a relative within-drug response measure. This calculation was the signed deviation of an observed AUC value from the mean AUC for that drug across FRT–CA, normalized to the mean AUC value. The new ‘Divergence AUC’ scores allow for a direct comparison of performance across FRT–CA combinations with the within-drug response variance now normalized.

### 2.6 Pathway analysis

Features selected in the RCDML process, genes with count data, were uploaded into cystoscape (v3.9; Shannon *et al.*, 2003) or the Molecular Signature Database (v7.5.1) for analysis (Subramanian *et al.*, 2005). Networks were analyzed and filtered (*P*-value < 0.01) based on their enrichment score (Fisher’s exact test) generated in the reactomeFI application for cytoscape (Gillespie *et al.*, 2022).

## 3 Results

Using the RNA-seq data and *ex vivo* drug response results from the BeatAML initiative, subjects were divided into responders versus non-responders using the lower and upper quartile approach (Section 2.2) for 24 RTK-type III inhibitors. Per each inhibitor, a 5-fold iterative approach was performed to optimize and select the RCDML models with highest AUC scores for 80% of the dataset. The best-performing models for each inhibitor were then used to calculate inference on the other 20% (holdout dataset). The full workflow is performed 10 times and the average of the sensitivity and specificity results for the holdout dataset are analyzed below.

### 3.1 Model performance

**Fig. 3.**
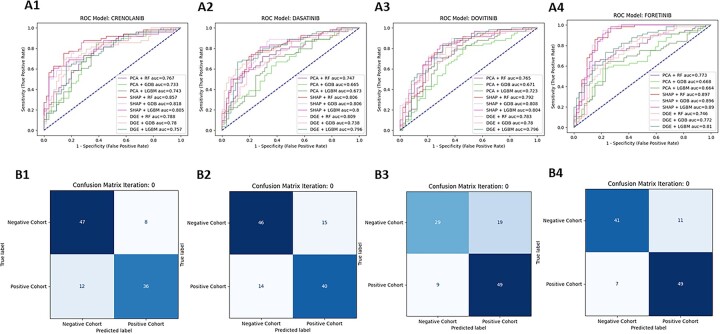
ROC–AUC curves and CM (SHAP + RF) for the top 4 RTK-type-III Inhibitor models. The top 4 performing RTK-type-III inhibitor models are assigned to a number as follows (1—Crenolanib, 2—Dasatinib, 3—Dovitinib, 4—Foretinib) (**A**) For each inhibitor, a ROC-AUC plot was generated. The ROC-AUC plots consist of ROC curves for nine different feature selection + classification combinations. The baseline (50% AUC) is represented with a blue-dashed line. For all the plots, the *x*-axis is 1—specificity, also called the false positive rate or fall-out and the *y*-axis is the sensitivity, also known as true positive rate or recall. The performance for each given model varies depending on the inhibitor chosen. (**B**) For each inhibitor, a CM plot was generated using the predicted outcomes from the SHAP + RF model combination. The CM plots consist of (Read from left to right, from top to bottom) the true negative, false negative, false positive and true positive values calculated by comparing the predicted classification assignments (*y*-axis, ‘Predicted Label’) versus the true classification assignments (*x*-axis, ‘True Label’). The squares of the CM plots are color coded in blue, where the shade of the color changes depending on the proportion of the samples that fall in the square (darker = more samples, lighter = less samples

**Fig. 4.**
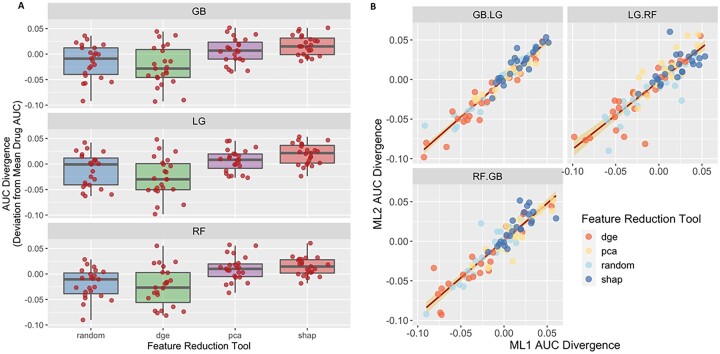
Evaluation of model performance. (**A**) Box-whisker plots for XGBoost (GB, top panel), LightGBM (LG, middle panel) and random forest (RF, bottom panel) were generated per each feature selection technique (PCA blue, DGE green, SHAP purple and random red) tested. The *x*-axis values are the AUC divergence score for the given feature selection + classifier combination of the 24 RTK-type-III inhibitors (red dots). (**B**) A linear regression was conducted to compare classifier 1 (*x*-axis) versus classifier 2 (*y*-axis) for all 24 RTK-TYPE-III inhibitors tested. The AUC scores for the 24 RTK-type-III inhibitors are plotted in a scatter plot, where the scores for one classifier are plotted against the scores of another classifier. The points in the graph are color coded to represent the different feature selection techniques according to the legend in the top left corner of the graph. A red line is plotted across the graph to show the correlation between the two models, with the variance plotted along the line as a yellow overlay. We see that there is a high correlation between the models, where the slope is not significantly different from 1 and the Intercept is not sig different from 0

**Fig. 5.**
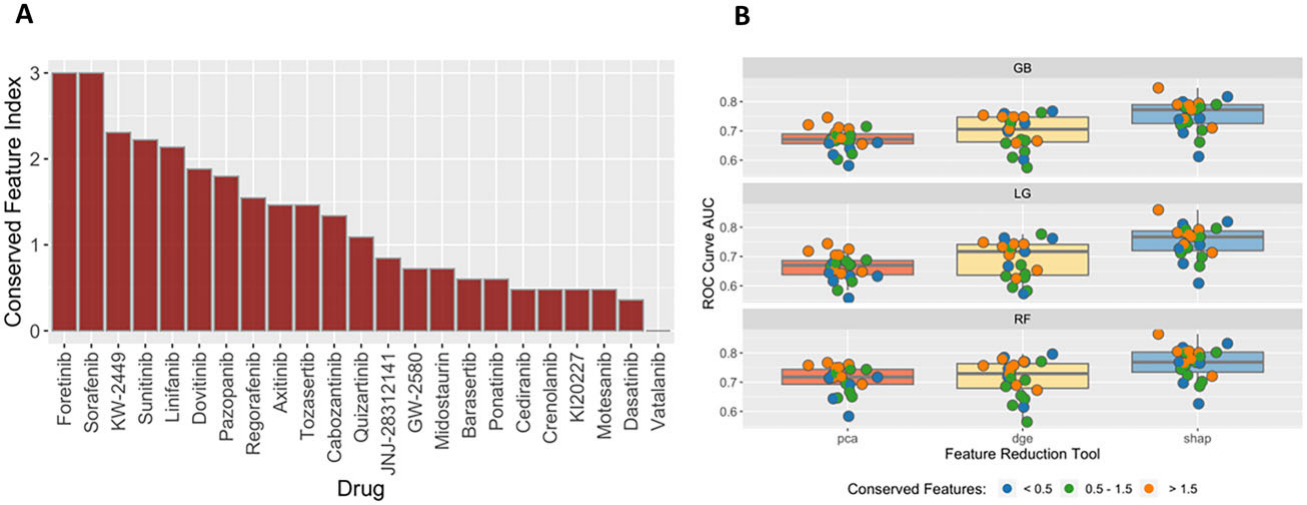
Analysis of feature conversation and model performance. (**A**) This plot shows the conservation index of the 24 RTK-type-III Inhibitor models. The *y*-axis corresponds to the conservation index value, whereas the *x*-axis corresponds to each of the 24 inhibitors. The conservation index value is calculated by taking the product of the feature counts divided by the sum squared. The higher the index value means the more features are shared across pipeline iterations. A low index value means that the features found in each fold are more unique and overlap less with the other folds. (**B**) A box-whisker plot for each feature selection tool (PCA red, DGE yellow and SHAP blue) was plotted for each model, top panel GB, middle panel LG and bottom panel RF. The random tool is omitted from this set of results because the features are assigned at random at each iteration so there is no conservation. Each box-whisker was generated using the AUC score for the given feature selection + classifier combination of the 24 RTK-type-III inhibitors. These scores are color coded (blue, green and orange) based on separating the conservation index (**A**) into three categorical bins: <0.5, 0.5–1.5 and >1.5

To evaluate the performance of the models tested (*n* = 9 per inhibitor), ROC curves were plotted for each inhibitor (Fig. 3A), as well as confusion matrices (CM) for each model combination (Fig. 3B) at the CV stage. The highest performing model was for Foretinib with 89.73% AUC using the SHAP technique combined with the RF classifier, followed by Dovitinib, KW-2449, Crenolanib and Dasatinib. For Foretinib, the false positive was 11 and the false negative was 7, and the true positive 49 and true negative was 41 (Fig. 3B4). Average of AUC, sensitivity and specificity of all RTK-type-III inhibitors can be found in Supplementary Table S6. The ROC–AUC curves and confusion matrices are available in Supplementary Figure S1. To compare the overall performance of these models and determine which approach was on average the most accurate, a ‘Divergence AUC’ score was analyzed by standard ANOVA (after assumptions of normality and homogeneity of variance were verified) using the hold out stage results and the top performing models from the CV stage. This enabled a comparison of the assessment of the difference in computational performance of the FRT and CA (Fig. 4). Comparing the results from all 24 inhibitors, the SHAP feature selection technique (purple box-whisker; Fig. 5A) out-performed PCA, DGE and the random tool (ANOVA *P* < 0.0001 and Tukey HSD, *P* < 0.0001) regarding AUC for the ROC curves. When analyzing the classifier (Fig. 5B), no statistical difference in performance was found, indicating that the feature selection technique has the greatest impact on overall classification performance than the use of either the RF or Gradient Boost classifier algorithm.

Additionally, we noted that there was a large difference across drugs in the number of features that were conserved across all models within folds and replicates (Fig. 5A). We assessed the impact of conserved feature selection on model performance as determined by AUC (Fig. 5B). Using a dimensionless scaled measure of conservation, we assessed the number of drug AUC scores above and below the mean AUC for each FRT–CA contrast. The conserved index was divided into three bins (low, middle or high conservation). The top conservation bin ‘>1.5’ for each FRT–CA contrast had significantly higher drug AUC scores above versus below the median AUC **(**prop.test() for equal proportions (Ho: Pr[‘>0.5 above median’] = 0.50, *P* = 0.0127, *n* = 9).

### 3.2 Random feature experiment

We conducted an experiment where we selected 30 features randomly as the baseline, to show the validity of the feature selection tools. To do this, we used the python random package, generating 30 random integers from the range of the total number of genes available and selecting genes that correspond to those indexes. We plotted these results alongside the results generated for the other feature selection techniques as seen on Figure 4A. The random feature selection tool (Fig. 4A) diverges negatively regarding AUC performance within drug compared with the other results. This comparison reaffirms that feature selection techniques are important because they help the classifiers make more informative decisions at the time of making predictions.

### 3.3 Performance of models using features selected from non-RTK-type-III inhibitors

To further evaluate model performance and specificity, we conducted an experiment in which features were ‘swapped’ between the different ML models developed. We used the features from two inhibitors of the PI3K-AKT-MTOR family (GSK6900693 and TG100-115) to make predictions for the 24 RTK-type-III inhibitor models.

When using the GSK6900693 features, 20 of the 24 models underperformed compared with when used the features intended for the models’ inhibitor (the baseline results). When using the TG100–T150, 23 of the 24 models underperformed. For the cases where the ‘swapped’ model performed better than the baseline, these models did not originally have a strong accurate performance so it is possible that by providing a different predictive signal these results could vary.

The full set of results can be found in Supplementary Table S7.

### 3.4 Analysis of feature importance

To assess the features (genes) being utilized in the various models developed, we analyzed feature importance and the potential biology using three different methods: (i**)** Feature importance based on the frequency each feature is split on, (ii**)** Feature importance based on SHAP contribution and (iii**)** Pathway analysis of the features selected for the top performing inhibitor models.

#### 3.4.1 Feature importance based on the frequency each feature is split on and on SHAP contribution

**Fig. 6.**
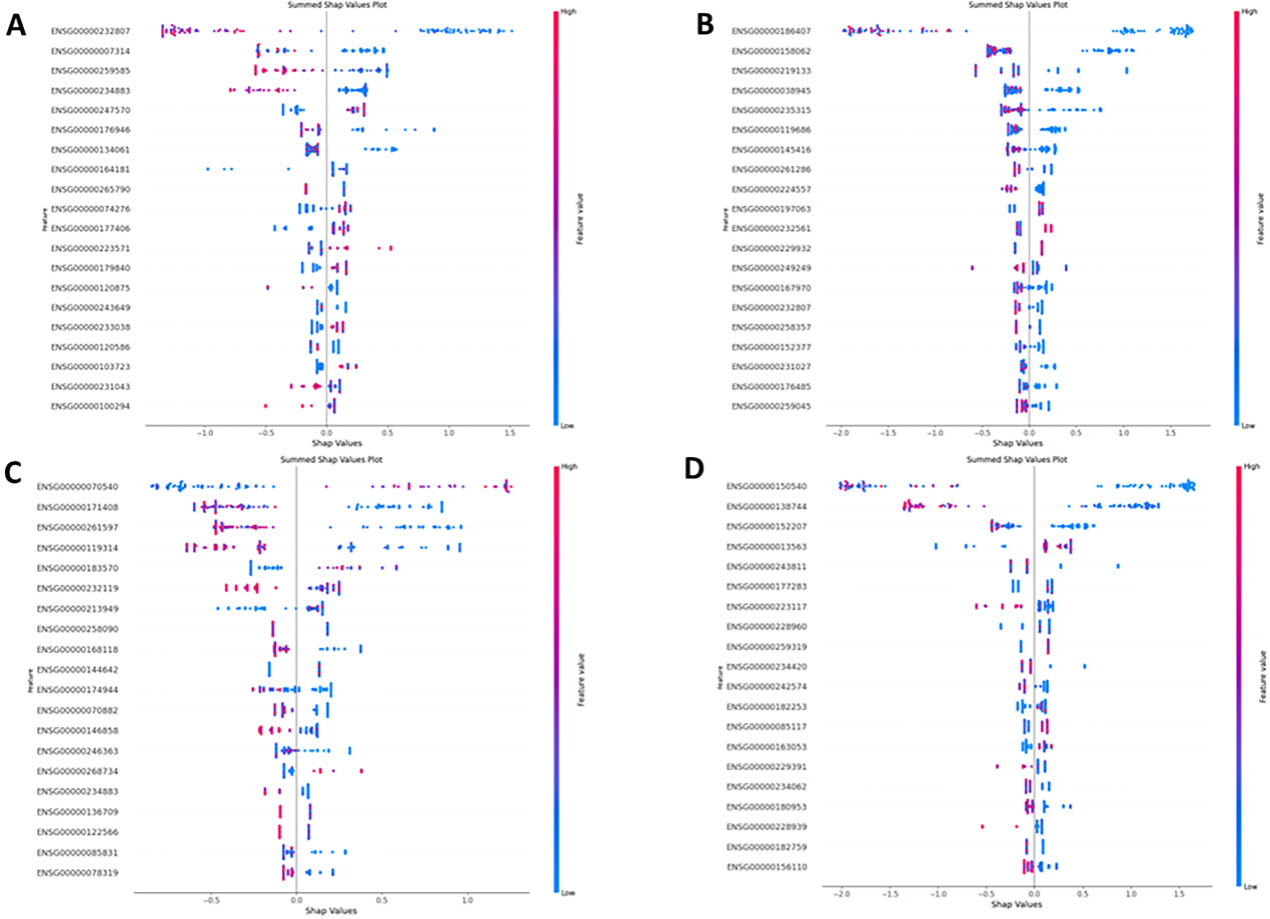
Feature importance plots for the top 4 performing RTK-type-III inhibitor models based SHAP contribution. The top 4 performing RTK-type-iii inhibitor models are assigned to a number as follows (**A**—Crenolanib, **B**—Dasatinib, **C**—Dovitinib and **D**—Foretinib). For each inhibitor a summed SHAP impact plot was generated. The y-label represents the model features ranked on largest summed SHAP value, from high to low. The *x*-axis represents the SHAP value score each observation has within a feature. Each observation is color coded based on the range of gene expression, where the closer the dot is to blue then the lower the gene expression value and the closer it is to red then the higher the gene expression value is

Features were ranked on how many times a specific feature gets split on as shown in Supplementary Figure S2, and on the overall impact a feature has based on their SHAP contribution value, as shown in Figure 6. From Figure 6, we can see that for each inhibitor there are three to five features that are shared between analyses (Fig. 6 and Supplementary Fig. S2) and have the strongest impact on the model. The rest of the features have a much lesser impact on the classification and seem to differ between runs. In the case for Crenolanib, Dasatinib and Foretinib, the top feature selected was the same for both analyses, whereas for Dovitinib the top feature found in the SHAP analysis (Fig. 6C) was third in the model feature importance plot (Supplementary Fig. 2c) and the top feature from this plot was the second in the SHAP analysis. The SHAP contribution plot (Fig. 6) gives us a bit more insight into how these features affected classification, as having a low feature value for the top feature in Crenolanib, Dasatinib and Foretinib has a strong correlation with being classified as a low responder, whereas it is the opposite for Dovinitib. By analyzing the model interpretability, we can identify strong candidates that can be evaluated for biological significance by performing a pathway analysis.

#### 3.4.2 Pathway analysis of features (genes) selected for predicting drug response

To evaluate the biology of the features (genes) selected for classification, a pathway analysis was conducted for 5 models out of the top 10 performing models: Dasatinib, Dovitinib, Foretinib, KW-2449 and Sorafenib. In total, there were 877 unique genes utilized in these models (Supplementary Table S8). Of those, 118 were assigned to a MSigDB gene family (Supplementary Table S9). In summary across the top 5 performing models, 21 of the key features were protein kinases, 1 were tumor suppressors, 14 were oncogenes, 13 translocated cancer genes, 14 cell differentiation markers, 36 transcription factors, 6 homeodomain proteins and 13 cytokines and growth factors (Supplementary Table S9).

Next, a pathway enrichment analysis was conducted for 5 models out of the top 10 performing models and are summarized in Table 2. Briefly, the features selected in the ML models were analyzed per drug. The top pathways were calculated and included: MHC **_*Class*_** II antigen presentation (dasatinib), nucleotide excision repair (dovitinib), g-protein signaling through tubby proteins (foretinib), Wnt signaling pathway (KW-2449) and CXCR3-mediatated signaling events (sorafenib). Of interest several of these pathways have been previously linked to drug resistance, including CXCR signaling for sorafenib (Ren *et al.*, 2020), supporting that our ML feature selection technique (SHAP) is capable of identifying and utilizing biological relevant gene features for classification. Additionally, several of the ML models consistently identified the same key features, including but not limited to HUS121 and FBXO522, which are well-established drug resistance markers.

**Table 2.** Pathway analysis of the top 5 RTK-type-III inhibitor models

Drug	Mode of action	Pathway identified from features	FDR-corrected *P*-value
Dasatinib	ATP-competitive inhibitor of SRC and ABL tyrosine kinase	MHC class II antigen presentation (Axelrod *et al.*, 2019)	6.91E-05
Dovitinib	Inhibits the phosphorylation of types III–V RTKs	Nucleotide excision repair (Duan *et al.*, 2020)	5.18E-03
Foretinib	Inhibits hepatocyte growth factor receptor c-MET and vascular endothelial growth factor receptor 2	g-protein signaling through tubby proteins	0.0105
Sorafenib	Multikinase inhibitor	CXCR3-mediated signaling events (Ren *et al.*, 2020)	8.21E-03
KW-2449	Multikinase inhibitor	Wnt signaling pathway (Chu and Small, 2009; Yu *et al.*, 2021)	0.0197

## 4 Discussion

The utilization of large-scale publicly available datasets is essential in the quest to improve the outcomes for cancer patients. In this project, we have leveraged an extensive resource established by the LLS, the BeatAML initiative (Leukemia and Lymphoma Society, 2021). Our work demonstrates that a robust feature selection technique coupled with a classifier can predict whether a sample will respond *ex vivo* to a given therapy with high accuracy. The extensive models tested support that the feature selection technique, moreso than the classifier, has a significant effect on model performance. Collectively, we applied a novel application of SHAP values as a feature selection process with genomic data.

In other research methods, SHAP has been applied after the training phase to examine what was leveraged in a given model. Instead, in our work, we apply the SHAP technique before the final training phase and use it as a feature selection technique. The reasons to do so include: (i) the high dimensionality of genomic data (feature size > sample size) that makes it a challenge to train certain classifiers due to overfitting/the curse of dimensionality, (ii) by reducing the number of features passed to the classifier we also reduce training time, (iii) we retain the ability to analyze the biological relevance of features used in the models. In our approach, we are determining a patient’s response to a drug therapy as a classification problem. By making this, a classification problem instead of a regression problem, new patients whose drug responses are unknown, could be assigned/classified (responder/non-responder).

The approach developed for this project could potentially be leveraged in real-time at the bedside at time of diagnosis, with a transcriptional profile being used to determine the best treatment options. Of interest, the pathway enrichment analysis of the top features selected (genes) indicated several well-known molecules, like HUS1, and signaling molecules like ERK as having the potential at time of diagnosis to indicate whether a patient will respond to a given drug. This type of analysis and data may help drive the identification of new therapeutic drug targets.

Future work that could improve performance of the models would be to expose the existing model to clinical or other types of data; however, the availability of such data is not common in the publicly available domain, as is the case for the BeatAML initiative used in this article.

In conclusion, this effort provides a comprehensive RNAseq Count Drug Response Machine Learning (*RCDML*) workflow that uses publicly available data, BeatAML, to classify patients as responder/non-responder to a given drug therapy. The ML framework is publicly available, https://github.com/UD-CRPL/RCDML, and can be used agnostically across various projects with similar data inputs. Collectively, the results and the divergence scores gave insights into the combination of a given feature selection + classifier combination, with these combinations being applied across 24 RTK-TYPE-III inhibitors, and demonstrated that a feature selection technique, in our case the SHAP technique, rather than the classifier in related work, has the greatest impact on model performance.

## Supplementary Material

vbad034_Supplementary_Data

## Data Availability

The dataset utilized for this project was provided in the original manuscript (Burd *et al*., 2020) and on the supplementary material, consisting of the RNA-seq count data (Supplementary Table S1) and ex vivo drug response data (Supplementary Table S2).
