# Supplementary material for: Machine learning classifier approaches for predicting response to RTK-type-III inhibitors demonstrate high accuracy using transcriptomic signatures and ex vivo data
Source: Bioinform Adv. 2023 Mar 22;3(1):vbad034. doi: 10.1093/bioadv/vbad034 (PMC10209528; doi:10.1093/bioadv/vbad034)
Supplement: vbad034_Supplementary_Data [file vbad034_supplementary_data.zip › Supplemental_Information_Final.docx]

**Supplementary Table 1. CPM RNASeq Data Matrix used from the BeatAML dataset**

Table is available in manuscript <https://www.nature.com/articles/s41586-018-0623-z>. Please download Supplementary Tables in the bottom of the manuscript and refer to Supplementary Table S9-Gene Counts CPM.

**Supplementary Table 2. Drug Response Data Matrix used from the BeatAML dataset**

Available as an excel file called: BeatAML_drug_response.xlsx

**Supplementary Table 3. Label assignments (responder vs non-responder) for the 24 RTK-Type-III Inhibitor Models**

Available as an excel file called: RTK_TYPE_III_balance_check.xlsx

**Supplementary Table 4. Drug Families Data Matrix used from the BeatAML dataset**

Available as an excel file called: BeatAML_drug_families.xlsx

**Supplementary Table 5. Python packages and version used to run the RCDML pipeline experiments**

Package list and versions:

| **Package** | **Version** | **Package** | **Version** |
| --- | --- | --- | --- |
| pandas | 1.3.5 | seaborn | 0.11.2 |
| xgboost | 1.5.2 | scikit-learn | 1.0.2 |
| matplotlib-venn | 0.11.6 | shap | 0.39.0 |
| statsmodels | 0.12.2 | xlrd | 2.0.1 |
| openpyxl | 3.0.9 | Python | 3.7.11 |
| pathlib | 1.0.1 | matplotlib | 3.5.0 |
| LightGBM | 3.3.2 |  |  |

**Supplementary Table 6. Average model performance results (AUC, sensitivity, specificity) for all model combinations (Feature Reduction Tool + Random Forest, Gradient Boosting) of 10 iterations of the 24 RTK-Type-III Inhibitor models**

Available as an excel file called: all_results_avg_REVISED.xlsx

**Supplementary Table 7. Performance Results of Models using Features Selected from non-RTK-type-III Inhibitors**

Available as an excel file called: swapped_features_results.xlsx

**Supplementary Table 8. Genes selected by the SHAP feature selection technique over 10 iterations for the Top 5 RTK-Type-III Inhibitor Models (Dasatinib, Foretinib, Dovitinib, KW-2449, Sorafenib)**

Available as an excel file called: SHAP_genes_selected.xlsx

**Supplementary Table 9. Summary of features used in the top performing models**

|  | Cytokines and  growth  factors | Transcription factors | Homeodomain  proteins | Cell  differentiation markers | Protein  kinases | Translocated  cancer genes | Oncogenes | Tumor  suppressors |
| --- | --- | --- | --- | --- | --- | --- | --- | --- |
| Tumor suppressors | 0 | 0 | 0 | 0 | 0 | 0 | 0 | 1 |
| oncogenes | 1 | 5 | 1 | 2 | 1 | 13 | 14 |  |
| Translocated cancer genes | 1 | 5 | 1 | 2 | 1 | 13 |  |  |
| Protein kinases | 1 | 1 | 0 | 2 | 21 |  |  |  |
| Cell differentiation  markers | 1 | 0 | 0 | 14 |  |  |  |  |
| Homeodomain proteins | 0 | 6 | 6 |  |  |  |  |  |
| Transcription factors | 0 | 36 |  |  |  |  |  |  |
| Cytokines and growth  factor | 13 |  |  |  |  |  |  |  |

**Supplemental Figure 1. Evaluation of Model Performance for 20 RTK-Type-III Inhibitors not included in the Main Manuscript**


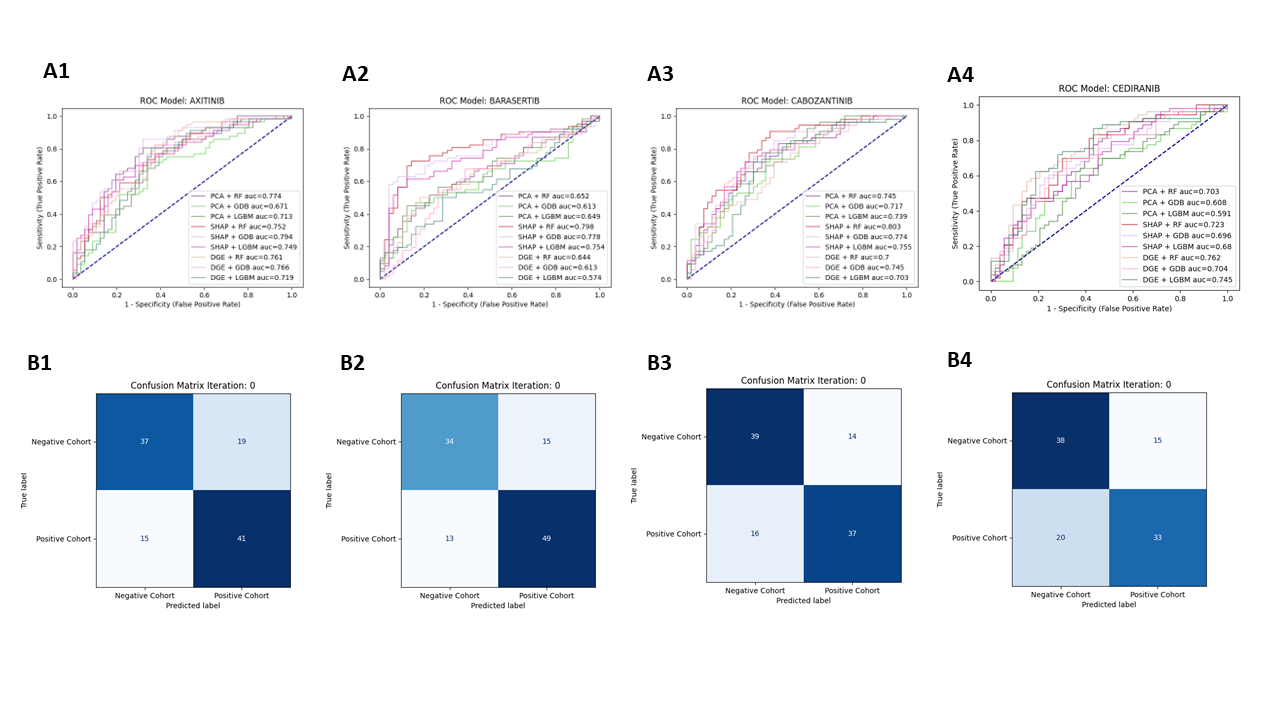


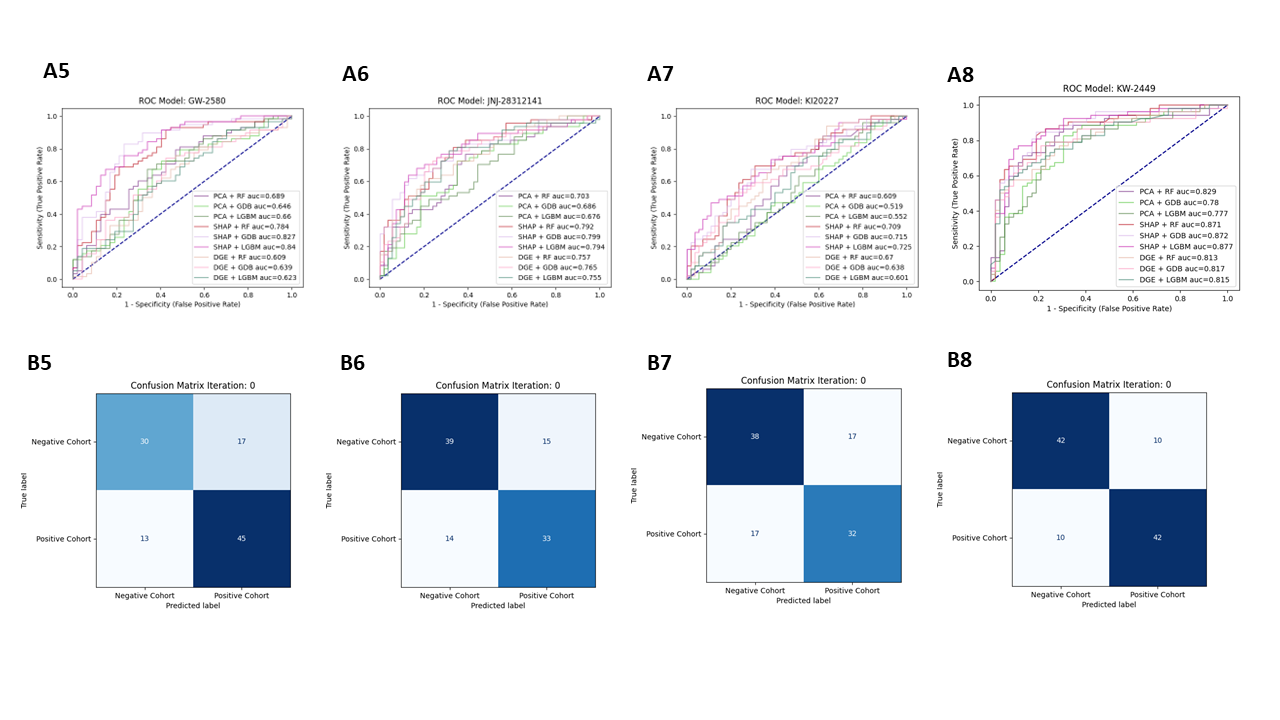


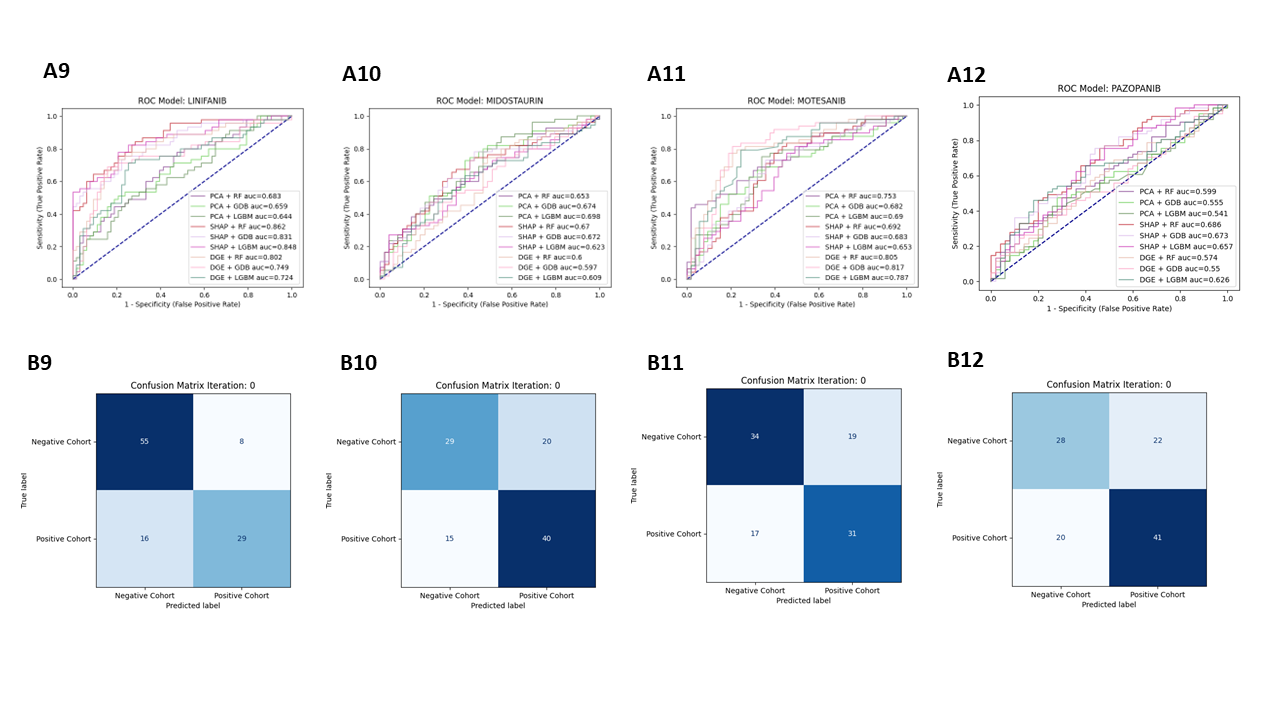


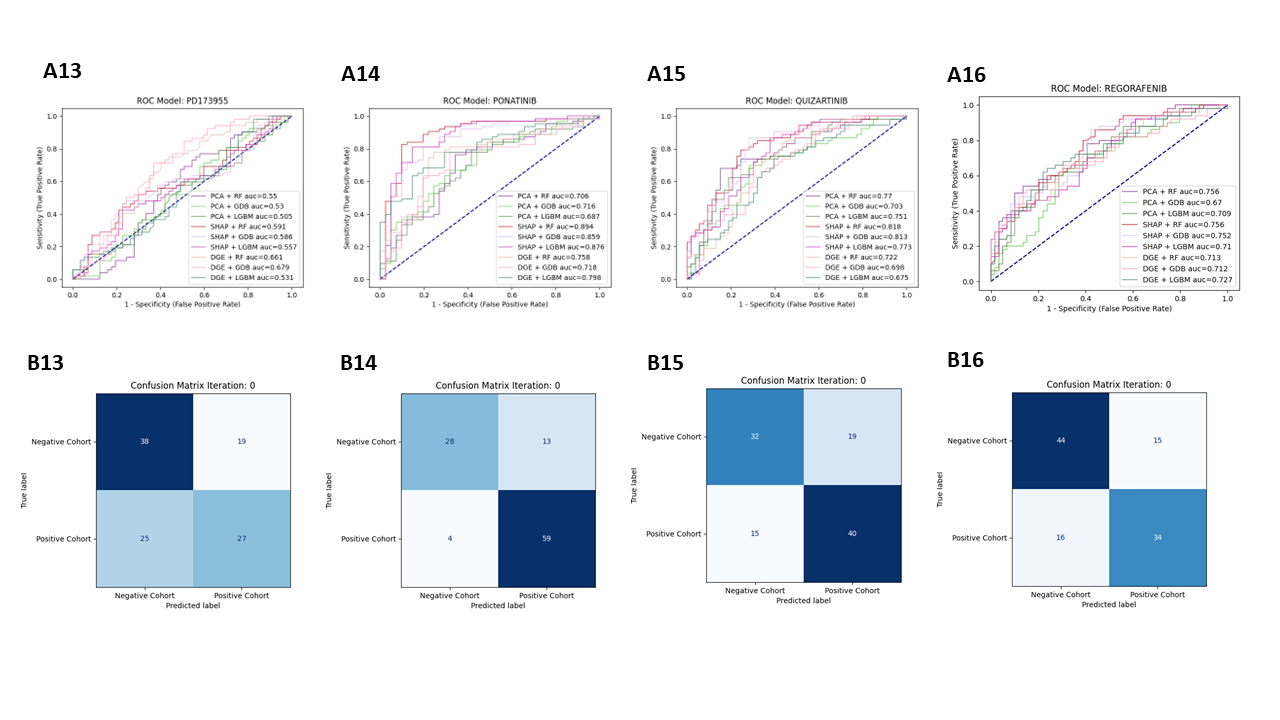


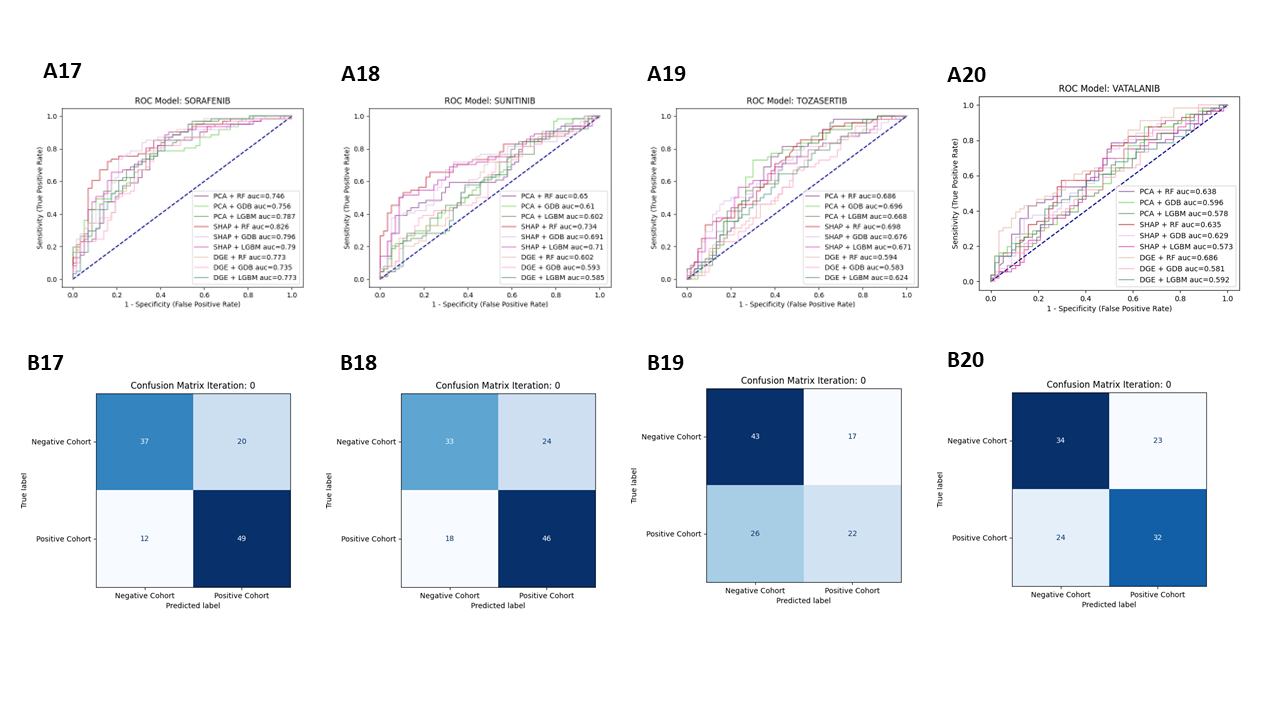


**Panel A:** For each inhibitor a ROC-AUC plot was generated. The ROC-AUC plots consist of ROC curves for 9 different feature selection + classification combinations. The baseline (50% AUC) is represented with a blue dashed line. For all the plots, the x-axis is 1 – Specificity, also called the False Positive Rate (FPR) or fall-out and the y-axis is the Sensitivity, also known as True Positive Rate (TPR) or recall. The performance for each given model varies depending on the inhibitor chosen. **Panel B:** For each inhibitor a Confusion Matrix (CM) plot was generated using the predicted outcomes from the SHAP + RF model combination. The CM plots consists of (Read from left to right, from top to bottom) the True Negative (TN), False Negative (FN), False Positive (FP), True Positive (TP) values calculated by comparing the predicted classification assignments (y-axis, “Predicted Label”) versus the true classification assignments (x-axis, “True Label”). The squares of the CM plots are color coded in blue, where the shade of the color changes depending on the proportion of the samples that fall in the square (darker = more samples, lighter = less samples

**Supplemental Figure 2. Feature importance plots for the top 4 performing RTK-type-iii inhibitor models based on feature split.**

**
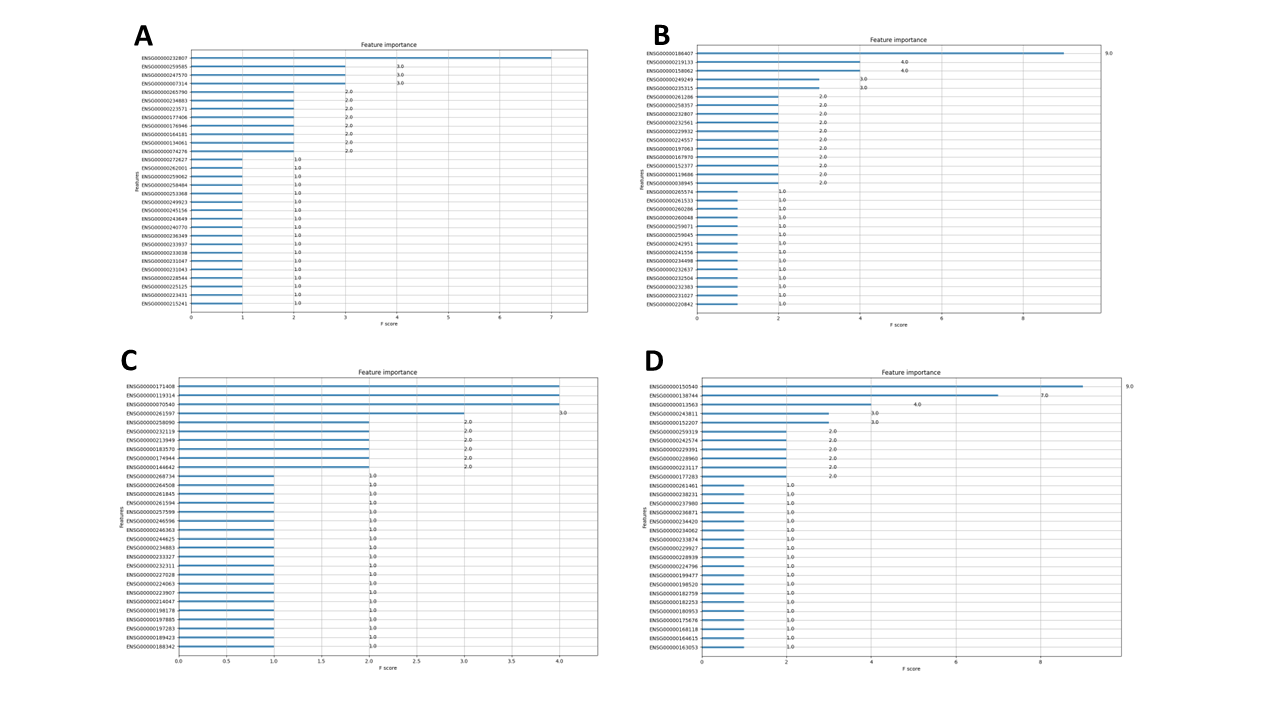
**

The top 4 performing RTK-type-iii inhibitor models are assigned to a number as follows (A - Crenolanib, B - Dasatinib, C - Dovitinib, D - Foretinib). A feature importance plot was generated for each inhibitor. The y-axis represents the model features ranked on largest F-score, high-to-low. The x-axis represents the F-score, or the number of how many times a feature was split on when forming the decision trees.
